# The impact of entering poverty on the unmet medical needs of Korean adults: a 5-year cohort study

**DOI:** 10.1186/s12889-022-14251-0

**Published:** 2022-10-07

**Authors:** Yun Hwa Jung, Sung Hoon Jeong, Eun-Cheol Park, Sung-In Jang

**Affiliations:** 1 Department of Public Health, Graduate School, Yonsei University, Seoul, Republic of Korea; 2 Institute of Health Services Research, Yonsei University, 50 Yonsei-ro, Seodaemun-gu, Seoul, 03722 Republic of Korea; 3 Department of Preventive Medicine, Yonsei University College of Medicine, Seoul, Republic of Korea

**Keywords:** Entering poverty, Unmet medical needs, Health care accessibility, Adult, Longitudinal

## Abstract

**Background:**

Studies on the effects of poverty on unmet medical needs are limited. Therefore, this study aimed to identify the impact of entering poverty on the unmet medical needs of South Korean adults.

**Methods:**

This study used data from the Korea Health Panel Survey (2014–2018) and included 10,644 adults. Logistic regression was used to examine the impact of entering poverty on unmet medical needs (poverty status: no → no, yes → no, no → yes, yes → yes; unmet medical needs: no, yes). Poverty line was considered to be below 50% of the median income.

**Results:**

When entering poverty, the proportion of unmet medical needs was 22.8% (adjusted odds ratio [AOR] 1.17, 95% confidence interval [CI] 1.01–1.36). Men (AOR 1.29, 95% CI 1.02–1.64), rural dwellers (AOR 1.24, 95% CI 1.01–1.50), and national health insurance (NHI) beneficiaries (AOR 1.21, 95% CI 1.04–1.42) were susceptible to unmet medical needs and entering poverty. Poverty line with below-median 40% had an AOR of 1.48 (95% CI 1.28–1.71). For the cause of unmet medical needs, the AORs were 1.50 for poverty (95% CI 1.16–1.94) and 1.08 for low accessibility to health care and information (95% CI 0.79–1.48).

**Conclusions:**

Entering poverty had the potential to adversely affect unmet medical needs. Men, rural dwellers, and NHI beneficiaries were vulnerable to unmet medical needs after entering poverty. Rigid definitions of poverty and inaccessibility to health care and information increase the likelihood of unmet medical needs and poverty. Society must alleviate unmet medical needs due to the increase in the population entering poverty.

## Background

Appropriate health care is a fundamental human right, and ensuring this is an important goal for the international community [1]. To meet medical needs, institutional efforts to improve access to health care at the national level have gained significance [2].

In Korea, the health insurance system was introduced in 1977, which was expanded to cover the entire nation in 1989 [3]. In addition, since 1946, community health centers have been continuously established to improve public health care and provide access to medical care for the low-income category, with the number of regional public health institutions being 3,571 in 2020 [4].

One important indicator of access to health care is unmet medical needs [5]. Unmet medical needs are conditions wherein a medical need is not met and is defined in two ways. First, in terms of “necessity for medical use,” unmet medical needs are states in which medical services are desired or necessary but not received. Second, in terms of “satisfying necessary medical services,” unmet medical needs are states where medical services are not provided adequately [6]. Unmet medical needs can increase the severity of diseases or lead to complications due to delayed treatment [7].

The unmet need experience rate in Korea decreased from 14.7% in 2011 to 11.6% in 2017 [8]. This is due to the improvement in access to medical care. The number of medical personnel per 100,000 population and the number of hospital beds per 1,000 population in Korea has been increasing gradually from the 1990s to the 2020s. The number of medical personnel per 100,000 population was 887 in 2011 and 1,081 in 2017. The number of beds per 1,000 population was 11.3 in 2011 and 13.5 in 2017 [9]. Nevertheless, Korea still faces problems with public health coverage. The unmet need experience rates for care still tend to be higher in Korea than in other Organization for Economic Co-operation and Development (OECD) countries [10]. The proportion of government and compulsory insurance in the current health expenditure was 61.0%, the fourth-lowest among OECD countries (Mexico = 49.3%, Greece = 59.8%, Chile = 60.6%, OECD average = 74.1%). Moreover, Korea’s medical expenses are growing at the fastest rate among OECD countries [11].

Poverty is a major factor impeding access to health care. In previous studies, it was found that the low-income class experienced relatively more unmet medical needs than the other income classes [12, 13]. Among the various definitions of poverty, relative poverty is defined as a certain percentage of a society’s median income as the poverty line and it refers to the extent to which resources are scarce compared to the living conditions of others [14]. We have focused on the effect of relative poverty on unmet medical needs because it is used to measure the social inequality and relative deprivation.

The “entering poverty” group is a vulnerable, high-stress group with significantly increased social risks, and it must adapt to worsening socioeconomic status. The main factors leading to entering poverty are old age, childbirth, widowhood or divorce, unemployment, disability, and government policies [15]. Given that it is difficult to change these entry factors for poverty in a short time period, there is a risk of prolonged poverty or worsening poverty. Poverty can make earning a living difficult, while also leading to social and emotional problems, such as family disintegration, crime, and suicide. Health threats while entering poverty can lead to irreversible and fatal events. Moreover, studies on entering poverty are relatively scarce in the literature.

Therefore, this study aimed to understand the impact of entering poverty on unmet medical needs among adults. The hypothesis is that entering poverty affects the growth of unmet medical needs among adults.

## Methods

### Data

The research data were taken from a 5-year (2014–2018) Korea Health Panel Survey (KHPS). KHPS is a representative panel survey of Korea on health care systems and diseases. This survey data provides individual and household level statistics on basic socioeconomic, medical conditions, and medical use. This survey includes longitudinal data measured repeatedly every year and is conducted by the Korea Institute for Health and Social Affairs and National Health Insurance Corporation [16]. The survey method was a computer-assisted personal interviewing method in which researchers visit target households. This study did not require approval or prior consent from the Institutional Review Board. KHPS is a secondary dataset available in the public domain and its data is de-identified to maintain anonymity and patient confidentiality. This survey was approved by the Korean National Statistical Office (Approval No. 920012).

### Participants

This study used the ninth wave of KHPS in 2014 as a baseline (15,263 respondents). Among them, 3840 people who were under the age of 19 and 779 with missing data were excluded. A total of 10,644 respondents (4960 men and 5684 women) from the baseline 2014 data were included and analyzed.

### Variables

The variable of interest was entering poverty. Entering poverty is defined as the state of poverty change from being above the poverty line in the previous year to dipping below the poverty line in the present year. In the main analysis, the poverty line was calculated below 50% of the median income, which is, the most common criterion used by OECD countries. In the sub-analysis, the criteria for the poverty line were also analyzed for 40% of the median income (strict poverty line) and 60% of the median income (used in the European Union [EU]) [17]. The entering poverty was analyzed by participants for each year if there was any change compared to the previous year, starting with the change in entering poverty in 2014 compared to 2013.

The main dependent variable was the presence or absence of unmet medical needs, and the sub-dependent variable was the cause of unmet medical needs. Unmet medical needs were measured as self-response to not receiving necessary hospital treatments or examinations in the past year. The causes of unmet medical needs were divided into “not unmet needs”, “mild symptoms”, “lack of time”, “low accessibility (distance or information)”, and “poverty”. Unmet medical care was analyzed by yearly participants from 2014 to 2018, the survey period.

Covariates were classified according to the concepts of predisposing, enabling, and need factors in the medical use model described by Andersen [7]. The predisposing factors included sex, age, marital status, educational level, and exercise. The enabling factors included region, economic activity, household income, and type of health insurance. The need factors included presence of chronic diseases, smoking, and drinking. Type of health insurance was categorized as national health insurance (NHI) (97% of the population) and medical aid (MA) (3% of the population) [18]. NHI is for nationals residing in Korea, excluding MA beneficiaries. NHI provides insurance benefits for disease prevention, diagnosis, treatment, rehabilitation, childbirth, death, and health promotion. MA is a public assistance service that the government provides for low-income people.

### Statistical analysis

Chi-square tests were conducted to analyze baseline characteristics according to the unmet medical needs among adults. A generalized estimating equation (GEE) model using PROC GENMOD with weight for analysis was conducted to evaluate the impact of poverty status on unmet medical needs. Subgroup analysis was stratified by sex, region, presence of chronic disease, and type of health insurance. Subgroup analyses were also conducted according to the type of poverty line and the causes of unmet medical needs. Results included adjusted odds ratios (AORs) and 95% confidence intervals (95% CIs). A *p* value ≤ 0.05 was considered statistically significant. Statistical analysis was performed using SAS®, version 9.4 (SAS Institute Inc., Cary, NC).

## Results

Table 1 presents the general characteristics of the participants at baseline 2014. Among 10,644 adults, there were 4960 (46.6%) men and 5684 (53.4%) women. The mean age of men was 50.9 ± 16.9 years and that of women was 52.6 ± 17.7 years. The mean age of all the participants was 51.8 ± 17.3 years. The proportion of unmet medical needs increased in the order of poverty status: no → no of (11.5%), yes → no (17.8%), no → yes (22.8%), and yes → yes (24.4%).

**Table 1:** Baseline characteristics of the study population (baseline 2014)

Variables	Unmet medical needs						
	**Total**		**Yes**		**No**		***P*-value**
	**N**	**%**	**N**	**%**	**N**	**%**	
**Total (*N* = 10,644)**	10,644	100.0	1,421	13.4	9,223	86.6	
**Poverty status (2013 → 2014)**							< 0.0001
No → No	8,866	83.3	1,024	11.5	7,842	88.5	
Yes → No	422	4.0	75	17.8	347	82.2	
No → Yes	536	5.0	122	22.8	414	77.2	
Yes → Yes	820	7.7	200	24.4	620	75.6	
**Sex**							< 0.0001
Men	4,960	46.6	579	11.7	4,381	88.3	
Women	5,684	53.4	842	14.8	4,842	85.2	
**Age**							< 0.0001
19–29	1,288	12.1	91	7.1	1,197	92.9	
30–49	3,704	34.8	437	11.8	3,267	88.2	
50–64	2,718	25.5	365	13.4	2,353	86.6	
65-	2,934	27.6	528	18.0	2,406	82.0	
**Region**							0.0167
Urban area	4,630	43.5	576	12.4	4,054	87.6	
Rural area	6,014	56.5	845	14.1	5,169	85.9	
**Marital status**							0.0111
Married	7,295	68.5	932	12.8	6,363	87.2	
Single, widow, divorced, separated	3,349	31.5	489	14.6	2,860	85.4	
**Educational level**							< 0.0001
College or over	3,924	36.9	373	9.5	3,551	90.5	
High school or below	6,720	63.1	1,048	15.6	5,672	84.4	
**Economic activity**							0.9669
Active	6,391	60.0	852	13.3	5,539	86.7	
Non-active	4,253	40.0	569	13.4	3,684	86.6	
**Household income**							< 0.0001
High	2,739	25.7	284	10.4	2,455	89.6	
Mid-high	2,614	24.6	285	10.9	2,329	89.1	
Mid-low	2,586	24.3	329	12.7	2,257	87.3	
Low	2,705	25.4	523	19.3	2,182	80.7	
**Smoking**							0.0714
No	8,450	79.4	1,102	13.0	7,348	87.0	
Yes	2,194	20.6	319	14.5	1,875	85.5	
**Drinking**							0.4561
Less	7,389	69.4	999	13.5	6,390	86.5	
Much	3,255	30.6	422	13.0	2,833	87.0	
**Practicing aerobic exercise**							0.0041
Yes	4,261	40.0	519	12.2	3,742	87.8	
No	6,383	60.0	902	14.1	5,481	85.9	
**Having chronic diseases**							< 0.0001
No	8,049	75.6	989	12.3	7,060	87.7	
Yes	2,595	24.4	432	16.6	2,163	83.4	
**Health insurance**							< 0.0001
National health insurance	10,263	96.4	1,316	12.8	8,947	87.2	
Medical aid	381	3.6	105	27.6	276	72.4	

Regarding poverty status, the poverty line in this table is based on less than 50% of the median income

Table 2 indicates the results of the analysis of factors concerning unmet medical needs using GEE analysis. Adults with unmet medical needs were found to have progressively higher AORs when exposed to persistent poverty compared to no poverty (poverty → no poverty: AOR 1.15, 95% CI 0.99–1.33; no poverty → poverty: AOR 1.17, 95% CI 1.01–1.36; poverty → poverty: AOR 1.44, 95% CI 1.25–1.65).

**Table 2:** Generalized linear model through GEE analysis with the unmet medical needs

Variables	Unmet medical needs	
	**AOR**	**95% CI**
**Poverty status**		
No → No	1.00	
Yes → No	1.15	(0.99–1.33)
No → Yes	1.17	(1.01–1.36)
Yes → Yes	1.44	(1.25–1.65)
**Sex**		
Men	1.00	
Women	1.40	(1.28–1.53)
**Age**		
19–29	1.00	
30–49	1.70	(1.46–1.96)
50–64	1.79	(1.53–2.10)
65-	1.90	(1.61–2.23)
**Region**		
Urban area	1.00	
Rural area	1.00	(0.93–1.07)
**Marital status**		
Married	1.00	
Single, widow, divorced, separated	1.22	(1.12–1.32)
**Educational level**		
College or over	1.00	
High school or below	1.14	(1.04–1.25)
**Economic activity**		
Active	1.00	
Non-active	0.77	(0.72–0.83)
**Household income**		
High	1.00	
Mid-high	1.09	(0.99–1.20)
Mid-low	1.22	(1.10–1.35)
Low	1.40	(1.23–1.59)
**Smoking**		
No	1.00	
Yes	1.28	(1.16–1.42)
**Drinking**		
Less	1.00	
Much	1.10	(1.02–1.20)
**Practicing aerobic exercise**		
Yes	1.00	
No	0.93	(0.87–1.00)
**Having chronic diseases**		
No	1.00	
Yes	1.03	(0.94–1.13)
**Health insurance**		
National health insurance	1.00	
Medical aid	1.65	(1.40–1.94)

Regarding poverty status, the poverty line in this table is based on less than 50% of the median income

Table 3 shows subgroup analysis of independent variables using GEE analysis for unmet health care needs. Furthermore, a subgroup analysis was performed for independent variables associated with entering poverty. (men: AOR 1.29, 95% CI 1.02–1.64, women: AOR 1.07, 95% CI 0.87–1.30; rural dwellers: AOR 1.24, 95% CI 1.01–1.50, urban dwellers: AOR 1.07, 95% CI 0.84–1.37; NHI beneficiaries: AOR 1.21, 95% CI 1.04–1.42, MA beneficiaries: AOR 0.71, 95% CI 0.38–1.30) When entering poverty, men, rural dwellers, and NHI beneficiaries were more vulnerable to unmet medical needs than their counterparts who were women, urban dwellers, and MA beneficiaries.

**Table 3:** Subgroup analysis of independent variables using GEE analysis of the unmet medical needs

Variables	Unmet medical care						
	**Poverty status**						
	**No → No**	**Yes → No**		**No → Yes**		**Yes → Yes**	
	**AOR**	**AOR**	**95% CI**	**AOR**	**95% CI**	**AOR**	**95% CI**
**Sex**							
Men	1.00	1.28	(1.02–1.61)	1.29	(1.02–1.64)	1.73	(1.38–2.16)
Women	1.00	1.06	(0.87–1.28)	1.07	(0.87–1.30)	1.25	(1.050–1.50)
**Region**							
Urban area	1.00	1.12	(0.89–1.41)	1.07	(0.84–1.37)	1.30	(1.04–1.64)
Rural area	1.00	1.17	(0.97–1.41)	1.24	(1.01–1.50)	1.51	(1.27–1.80)
**Health insurance**							
National health insurance	1.00	1.18	(1.01–1.37)	1.21	(1.04–1.42)	1.44	(1.25–1.67)
Medical aid	1.00	0.98	(0.54–1.77)	0.71	(0.38–1.30)	0.97	(0.56–1.70)

Regarding poverty status, the poverty line in this table is based on less than 50% of the median income. The adjusted covariates are age, marital status, educational level, economic activity, household income, smoking, drinking, practicing aerobic exercise, and having chronic diseases

**Fig. 1:**
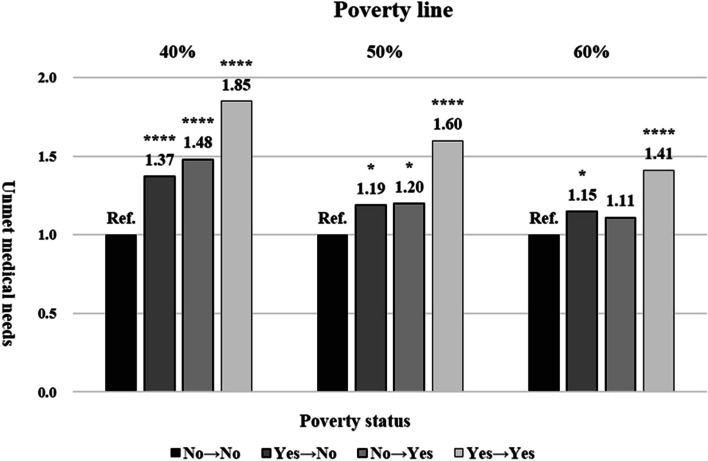
Generalized linear model through GEE with unmet medical needs by poverty status. The poverty lines in this figure are analyzed based on 40%, 50%, and 60% of median income, respectively. The adjusted covariates are sex, age, region, marital status, educational level, economic activity, household income, smoking, drinking, and practicing aerobic exercise, having chronic diseases, and health insurance. GEE = generalized estimating equations. Statistically significant *: *p* ≤ 0.05, **: *p* ≤ 0.01, ***: *p* ≤ 0.001, ****: *p* ≤ 0.0001. Ref. = Poverty status, No → No

Figure 1 presents the unmet medical needs by poverty status. Unmet medical needs were analyzed when the poverty line was 40% of median income (strict poverty line), 50% (most common and used by OECD countries) and 60% (used by the EU and OECD countries). Overall, the stricter the poverty line, the higher the AOR for unmet care tended to be. In the case of entering poverty, the AOR values ​​for unmet medical needs increased in the order of 60%, 50%, and 40% of median income (poverty line, 60% of median income: AOR 1.11, 95% CI 0.95–1.30, poverty line, 50%: AOR 1.20, 95% CI 1.03–1.40; poverty line, 40%: AOR 1.48, 95% CI 1.28–1.71).

**Fig. 2:**
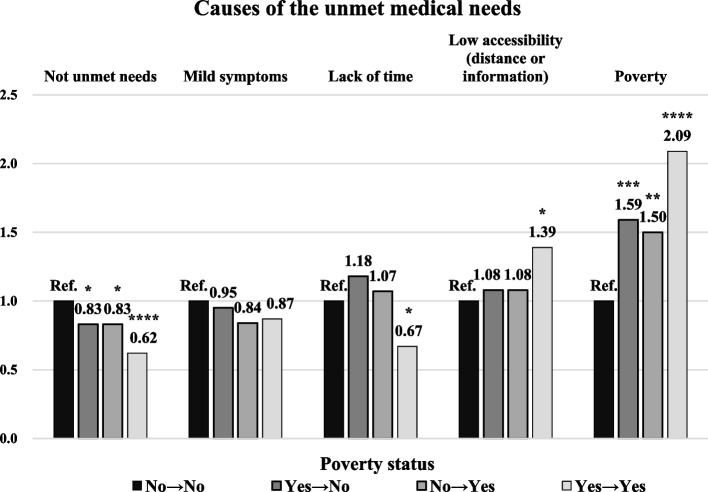
Generalized linear model through GEE with causes of the unmet medical needs by poverty status. The poverty line in this figure is based on less than 50% of the median income. The adjusted covariates are sex, age, region, marital status, educational level, economic activity, household income, smoking, drinking, and practicing aerobic exercise, having chronic diseases, and health insurance. GEE = generalized estimating equations. Statistically significant *: *p* ≤ 0.05, **: *p* ≤ 0.01, ***: *p* ≤ 0.001, ****: *p* ≤ 0.0001. Ref. = Poverty status, No → No

Figure 2 shows the analysis results of the causes of unmet medical needs by poverty status. The causes of unmet medical needs were analyzed in the presence of each cause compared to its absence, according to the poverty status. In the case of entering poverty compared to non-persistent poverty, AOR values for each cause of unmet medical needs were; 1.50 in poverty (95% CI 1.16–1.94), 1.08 in low accessibility (95% CI 0.79–1.48), 1.07 in lack of time (95% CI 0.78–1.47), 0.84 in mild symptoms (95% CI 0.62–1.12), and 0.83 in medical needs met (95% CI 0.71–0.96).

## Discussion

This study demonstrated the impact of entering poverty on the unmet medical needs of South Korean adults. Unmet medical needs tended to increase in the case of entering poverty based on the most common poverty line (50% of median income). The tendency was evident in the following cases: men, rural area, and NHI beneficiaries. At the strict poverty line (40% of median income), unmet medical needs were more pronounced in the entering poverty group. Poverty and low accessibility (distance or lack of information) were vulnerable factors among the causes of unmet medical needs.

In this 5-year panel survey, 13.4% of adults had unmet medical needs, which was consistent with the findings from a previous study [8]. The entering poverty group had approximately twice the prevalence of unmet medical needs compared with the persistently no-poverty group. They also had a higher risk of unmet medical needs than the poverty escape group. This may be because unmet medical needs are strongly affected by poverty, mainly focusing on current income levels.

Women were at risk for entering poverty and for unmet medical needs in previous studies [15, 19]. However, when entering poverty, men had a higher risk of unmet medical needs than women. In the strong psychological distress of entering poverty, this may be because women tend to relieve stress through social means such as chatting, while men have a greater tendency to relieve stress through alcohol and tobacco [20, 21]. Unhealthy behaviors such as alcohol and tobacco consumption can increase unmet medical needs for men entering poverty. Another reason may be that households in which men provide the main source of income are more focused on economic activities to escape from poverty and have less time to use health care.

By place of residence, rural residents are more vulnerable to unmet medical needs when entering poverty than urban residents. Most of all, it may be because access to medical care is inconvenient in rural areas. The proportion of rural villages with a community health center less than a 10-min drive away was 44.6% and that with a hospital or an oriental clinic was 31.6% [22]. In ri (village)-level administrative units, the proportion of areas operating six times a day or less was 40.0% [23, 24]. Entering poverty may not provide sufficient resources to overcome inconvenient access to health care. Rural areas can also exacerbate unmet medical needs because of the lack of jobs and relatively low incomes.

Korea is implementing a NHI system with the NHI (97% of the population) and MA (3% of the low-income class). When analyzing the impact of health insurance on unmet medical needs, MA beneficiaries were more likely to have unmet medical needs. However, in the case of entering poverty within each factor, the NHI beneficiaries were more vulnerable to unmet medical needs.

Medical benefit beneficiaries have a cost-sharing of 10% or less for hospitalization, and ₩2,000 ($1 = approximately ₩1120) or less or 15% or less for outpatient use [25]. Conversely, the NHI has a subdivided cost-sharing policy according to age, region, medical institution, and disease [26]. The out-of-pocket amount of the NHI is generally larger than MA. 10% of MA beneficiaries overuse health care, accounting for 60% of total MA expenditure [27]. In MA, the real value of health care decreases, mitigating the unmet health impact of entering poverty. NHI beneficiaries may be relatively vulnerable to unmet medical needs because there is no buffer for health care in the face of poverty. Depending on whether each country's health insurance behavior is affected by income, caution is needed in interpreting generalizations.

The criteria for the poverty line were analyzed as 40% (strict poverty line), 50% (most common poverty line, used in OECD), and 60% (poverty line used in EU) of median income, as follows: unmet medical needs risk from entering poverty: strict poverty line > the most common > used in EU. The gradual increase in unmet medical needs along the poverty line is likely an inference to suggest that the severity of poverty increases the risk of unmet medical needs when entering poverty.

When persistent non-poverty was the reference, the likelihood of unmet medical needs due to lack of money was in the following order: persistent poverty > escape from poverty > entering poverty. The likelihood of unmet medical needs due to low access was the next highest: persistent poverty > entering poverty = poverty escape. Since the risk of entering poverty is relatively high in “poverty” and “low access (distance or information)” of unmet medical needs causes, people at risk of entering poverty can consider material, physical, and information resources as necessary elements to alleviate unmet medical needs.

This study has some limitations. First, this study did not analyze the frequency or the types of medical use such as hospitalization, emergency, or outpatient treatment of unmet medical needs. In addition, we could not analyze disease groups and severity of unmet medical needs as there was no survey data. Second, it is not possible to know the expenses of loans or financial help from charities, public institutions, or acquaintances due to lack of data. Therefore, it is necessary to interpret the results carefully.

## Conclusions

When transitioning from no poverty to poverty, unmet medical needs are likely to increase. Men, rural dwellers, and NHI beneficiaries are tended to be vulnerable to unmet medical needs when entering poverty. More stringent the definition of poverty, the greater the risk of unmet medical needs. Appropriate policies are needed to mitigate the occurrence of unmet medical needs due to entering poverty.

## Data Availability

Publicly available datasets were analyzed in this study. These data can be found here: [https://www.khp.re.kr:444/web/data/data.do] (accessed on 12 January 2022).

## Abbreviations

AOR: Adjusted odds ratio
CI: Confidence interval
NHI: National health insurance
CHC: Community health centers
OECD: Organization for Economic Co-operation and Development
KHPS: Korea Health Panel Survey
EU: European Union
MA: Medical aid
GEE: Generalized estimating equation
NHI: National health insurance
